# Evaluation of the impact of continuous Kangaroo Mother Care (KMC) initiated immediately after birth compared to KMC initiated after stabilization in newborns with birth weight 1.0 to < 1.8 kg on neurodevelopmental outcomes: Protocol for a follow-up study

**DOI:** 10.1186/s13063-023-07192-5

**Published:** 2023-04-10

**Authors:** E. A. Adejuyigbe, I. Agyeman, P. Anand, H. C. Anyabolu, S. Arya, E. N. Assenga, S. Badhal, N. W. Brobby, H. K. Chellani, N. Chopra, P. K. Debata, Q. Dube, T. Dua, L. Gadama, R. Gera, C. K. Hammond, S. Jain, F. Kantumbiza, K. Kawaza, E. N. Kija, P. Lal, M. Mallewa, M. K. Manu, A. Mehta, T. Mhango, H. E. Naburi, S. Newton, I. Nyanor, P. A. Nyako, O. J. Oke, A. Patel, G. Phlange-Rhule, R. Sehgal, R. Singhal, N. Wadhwa, A. B. Yiadom

**Affiliations:** 1 Department of Paediatrics and Child Health, Obafemi Awolowo University, Ile-Ife, 220005 Nigeria; 2 Komfo Anokye Teaching Hospital, P.O. Box 1934, Adum, Kumasi, Ghana; 3 Department of Pediatrics, Vardhman Mahavir Medical College and Safdarjung Hospital, Ansari Nagar, New Delhi, 110029 India; 4 Department of Paediatrics and Child Health, School of Medicine, Muhimbili University of Health and Allied Sciences, Dar Es Salaam, 255 Tanzania; 5 Vardhman Mahavir Medical College and Safdarjung Hospital, Ansari Nagar, New Delhi, 110029 India; 6 Department of Child Health, Komfo Anokye Teaching Hospital, Kwame Nkrumah University of Science and Technology, Kumasi, Ghana; 7 Queen Elizabeth Central Hospital, Blantyre, Malawi; 8 Department of Mental Health and Substance Use, World Health Organization, Geneva, Switzerland; 9 Department of Obstetrics and Gynaecology, Kamuzu University of Health Sciences, Blantyre, Malawi; 10 Department of Paediatrics and Child Health, Kamuzu University of Health Sciences, Blantyre, Malawi; 11 Atal Bihari Vajpayee Institute of Medical Sciences &, Dr Ram Manohar Lohia Hospital, New Delhi, 110001 India; 12 School of Public Health, Kwame Nkrumah University of Science and Technology, Kumasi, Ghana; 13 Research and Development, Komfo Anokye Teaching Hospital, P.O. Box 1934, Adum, Kumasi, Ghana; 14 Department of Psychiatry, Child And Adolescent Mental Health, Komfo Anokye Teaching Hospital, P.O. Box 1934, Adum, Kumasi, Ghana; 15 Division of Epilepsy & Clinical Neurophysiology, Boston Children’s Hospital, Harvard Medical School, Boston, USA; 16 Clinical Development Services Agency (CDSA), Translational Health Science and Technology Institute (THSTI), NCR Biotech Science Cluster, PO Box #04, Faridabad, 121001 India; 17 Translational Health Science and Technology Institute (THSTI), NCR Biotech Science Cluster, PO Box #04, 121001 Faridabad, India; 18 Faridabad-Gurgaon Expressway, Translational Health Science and Technology Institute, NCR Biotech Science Cluster, 3Rd MilestonePost Box #04, Faridabad, Haryana 121001 India

**Keywords:** Immediate Kangaroo Mother Care (iKMC), Low-birth-weight babies, Neurodevelopment, Low- and middle-income countries

## Abstract

**Background:**

Preterm birth or low birth weight is the single largest cause of death in newborns, however this mortality can be reduced through newborn care interventions, including Kangaroo Mother Care (KMC). Previously, a multi-country randomized controlled trial, coordinated by the World Health Organization (WHO), reported a significant survival advantage with initiation of continuous KMC immediately after birth compared with initiation of continuous KMC a few days after birth when the baby is considered clinically stable.

Whether the survival advantage would lead to higher rates of neurodevelopmental morbidities, or the immediate KMC will also have a beneficial effect on cognitive development also, has not been investigated. We therefore propose to test the hypothesis that low-birth-weight infants exposed to immediate KMC will have lower rates of neurodevelopmental impairment in comparison to traditional KMC-treated infants, by prospectively following up infants already enrolled in the immediate KMC trial for the first 2 years of life, and assessing their growth and neurodevelopment.

**Methods:**

This prospective cohort study will enroll surviving neonates from the main WHO immediate KMC trial. The main trial as well as this follow-up study are being conducted in five low- and middle-income countries in South Asia and sub-Saharan Africa. The estimated sample size for comparison of the risk of neurodevelopmental impairment is a total of 2200 children. The primary outcome will include rates of cerebral palsy, hearing impairment, vision impairment, mental and motor development, and epilepsy and will be assessed by the age of 3 years. The analysis will be by intention to treat.

**Discussion:**

Immediate KMC can potentially reduce low-birth-weight-associated complications such as respiratory disease, hypothermia, hypoglycemia, and infection that can result in impaired neurocognitive development. Neuroprotection may also be mediated by improved physiological stabilization that may lead to better maturation of neural pathways, reduced risk of hypoxia, positive parental impact, improved sleep cycles, and improved stress responses. The present study will help in evaluating the overall impact of KMC by investigating the long-term effect on neurodevelopmental impairment in the survivors.

**Trial registration:**

Clinical Trials Registry-India CTRI/2019/11/021899. Registered on 06 November 2019.

Trials registration of parent trial: ACTRN12618001880235; Clinical Trials Registry-India: CTRI/2018/08/015369.

## Background

Nearly, 15.5% of all births, or more than 20 million infants worldwide, are born with low birth weight (LBW) as a result of preterm birth or intrauterine growth restriction or both [1, 2]. Preterm birth or LBW is the single largest cause of death in newborns, but the mortality can be reduced by effective newborn care interventions. However, for years, it was demonstrated that although mortality was being improved by interventions for LBW newborns, the impact on morbidity, including neurocognitive impairment and motor disabilities, was unchanged [3]. More recently, there is evidence suggesting that infant survival with improved neurodevelopmental outcomes is increasing slightly and is associated with both improved medical interventions and maternal factors [4]. However, there is also a concern of an increased risk of neurodevelopmental impairment, and this enigma needs to be addressed by a systematic study. Therefore, while evaluating the impact of any intervention on mortality in this population, it is equally important to study the risk of neurodevelopment impairments, including cerebral palsy, hearing and vision impairment, cognitive impairment, and epilepsy.

The World Health Organization (WHO) defines Kangaroo Mother Care (KMC) as a technique used in preterm and LBW infants of continuous skin-to-skin contact between mother and baby until the infant shows signs of intolerance. Exclusive breastfeeding is also encouraged as an essential component of KMC. Notably, however, it is currently recommended that continuous KMC should be initiated only once the baby is stable, meaning that the baby must be breathing spontaneously without additional oxygen [5].

“WHO recommendations on interventions to improve preterm birth outcomes” recommends KMC for the routine care of newborns weighing 2.0 kg or less at birth, which should be initiated in health care facilities as soon as the newborns are clinically stable [6]. These babies should be provided as close to continuous KMC as possible. Intermittent KMC, rather than conventional care, is recommended for newborns weighing 2.0 kg or less at birth, if continuous KMC is not possible [6]. There is no current recommendation for KMC for unstable < 2.0 kg neonates. A recently updated Cochrane review reported 40% lower mortality in infants with birth weight < 2.0 kg given KMC compared to mortality in those who were given standard care in hospitals, at 40 to 41 weeks postmenstrual age [7]. In almost all studies included in the Cochrane review, KMC was initiated after the baby was clinically stable, with the median age at initiation of KMC 3.2 to 24.5 days [7]. Two randomized controlled trials (RCTs) comparing immediate versus conventional KMC from South Africa and Vietnam [8–10] showed favorable results for immediate KMC initiation. This data led the World Health Organization to coordinate a multi-country randomized controlled trial to evaluate the effect of initiating continuous KMC immediately after birth on survival (compared with initiation of continuous KMC a few days after birth when the baby is considered clinically stable). This RCT assessed newborn survival outcomes over the acute hospital period up to a 28-day follow-up [11]. The study enrolled 3211 infant-mother dyads with 1609 dyads in the intervention arm and 1602 in the control arm. The median daily duration (hours) of skin-to-skin contact was 16.9 h (IQR 13.0–19.7) in the intervention and 1.5 h (IQR 0.3–3.3) in the control arm. Neonatal death occurred in 12.0% and 15.7% infants, respectively, in the two arms (RR 0.75; 95% CI 0.64–0.89; *p* = 0.001). The study concluded that in infants with birth weight between 1.0 and < 1.8 kg, immediate KMC (versus conventional KMC care) resulted in a significant reduction in neonatal mortality [12]. However, in order to evaluate the overall impact of KMC, the long-term effect on neurodevelopment in the survivors should be assessed.

It has been observed that KMC can reduce bradycardia and oxygen desaturation events in preterm infants, providing physiological stability and possible benefits for neurodevelopmental outcomes [13]. Few studies involving KMC initiated in stable infants show that KMC has a beneficial effect on both short-term [14] and long-term [15, 16] neurodevelopmental outcomes of preterm and LBW babies compared to no KMC. The long-lasting social and behavioral protective effects have been observed beyond 20 years after the KMC intervention [17].

Studies have suggested that there is a positive impact of KMC on parental interaction due to not only increased skin-to-skin contact but also increased opportunity for bonding through breastfeeding and, as a result, impact on the child’s behavior, which can be long lasting. However, the degree of direct impact on cognition, language, motor, auditory, and vision impairment has not been consistent, although most studies do demonstrate a positive trend in these domains [17–21]. Furthermore, emerging evidence from smaller studies suggests that, in addition to the positive parental impact, the implementation of KMC may also be neuroprotective through both improved sleep cycles and brain maturation patterns seen on EEG [22, 23], as well as improved stress responses in general, which had a lasting impact when reviewed over a 10-year period on both emotional and cognitive development [24].

Despite the evidence of the benefit of KMC after stabilization on neurodevelopmental outcomes, an evaluation of the long-term effects of the KMC initiated immediately after birth on the risk of neurodevelopmental impairment such as motor or sensory impairment or cognitive deficits is not well known.

We hypothesize that KMC initiated immediately after birth can improve physiological stabilization and potentially lead to anatomical or functional changes in the brain in the neonatal period, thus allowing for better maturation of the brain and pathways leading to a possible decrease in neurocognitive morbidity as well as reduce the risk of hypoxia, thus decreasing long-term morbidity. In addition, given the positive impact of traditional KMC on parental bonding, it is argued that the earlier KMC is initiated (e.g., with immediate KMC), the stronger the bond, leading to better family motivation and stimulation and thus positively impacting on child development.

The main outcome of interest for the parent study on immediate KMC was the impact on neonatal mortality [11, 12]. Evidence of the longer-term effect of the intervention on the risk of neurodevelopmental impairment, growth, and mortality beyond the neonatal period, on the other hand, will provide additional evidence and rationale for policy change as the intervention targets survival as well as helping babies to thrive.

We therefore propose to follow up the newborns enrolled in the recently published immediate KMC study [11, 12], beyond the neonatal period up to 3 years of age to additionally study the risk of neurodevelopmental impairments, specifically the risk on growth, feeding, caregiving practices, mortality, and home environment. Our main hypothesis is that those newborns who were provided continuous KMC initiated immediately after birth will experience a reduced risk of neurodevelopmental impairment, including the risk of cerebral palsy, hearing impairment, vision impairment, mental and motor impairments, and epilepsy, compared with a similar group in whom KMC was initiated only after stabilization.

## Methods

### Study design

This is a prospective cohort study of the children already enrolled in the immediate KMC randomized controlled trial to evaluate the impact of continuous Kangaroo Mother Care initiated immediately after birth (iKMC) on survival of newborns with birth weight between 1.0 and < 1.8 kg [11, 12].

### Study setting

As this study is a follow-up of the concluded and recently published iKMC trial [12], the study is being conducted at the same five LMIC study sites encompassing hospitals in Ghana, India, Malawi, Nigeria, and Tanzania. The selected facilities are tertiary care hospitals that care for small and sick newborns with a high proportion of LBW babies and follow-up high-risk newborns. As part of standardization across the involved study sites, prior to the study, trainings and standardization assessments were done to ensure all participating sites could offer the WHO minimum package of care for small and sick newborns [11].

### Study population

All infants born with birth weight between 1.0 and < 1.8 kg, enrolled in the main trial, who survive the neonatal period, and whose parents consent for their participation in the follow-up study, are eligible to be enrolled.

All infants who die within the first month of life or whose parents refuse consent for follow-up will be excluded from the study.

We employ two strategies to approach the parents for enrolment. First, we approach the mother/parent during the 29-day follow-up visit for the main trial. The second strategy is for children who have completed the 29-day follow-up visit in the main trial. For these children, we approach the mother/parent either telephonically or when they visit the high-risk infant follow-up clinic as part of standard hospital care. A verbal preconsent is taken and an in-person hospital visit is scheduled for a detailed written informed consent for participation in the follow-up study.

### Sample size calculation

The sample size for the main iKMC trial was a total of 4200 neonates. Early completion in January 2020 led to a total of 3211 neonates being enrolled in the main iKMC trial. A preliminary review of literature of neurodevelopment in infants between 1 and < 1.8 kg shows the prevalence of any of the conditions of cerebral palsy, hearing impairment, vision impairment, and mean development scores to vary from 5 to 25% [25–27]. Any neurodevelopmental impairment (e.g., cerebral palsy, Bayley score < 85, hearing or vision impairment) in the study population is estimated to be approximately at 15–25%. We estimated the sample size for comparison of the risk of neurodevelopmental impairment in control and intervention groups (20% compared with 15% or 25% [25% lower or higher]) with 80% power and a significance level of 5% to be 1100 per group, requiring a total of about 2200 children.

We thus propose to enroll 2200 babies from the main trial to reach the above-mentioned sample size.

### The intervention

The proposed study is a follow-up of the already concluded randomized controlled trial for evaluating the effect of immediate KMC on infant survival. The intervention and control groups were assigned during the main iKMC trial where KMC is defined as continuous skin-to-skin contact with mother or her surrogate aiming for at least 20 h per day, support for exclusive breastfeeding, and required medical care without separation from the mother as much as possible.

In the intervention group, the mother and baby remained in skin-to-skin contact from the time of randomization whereas the newborns randomized to the control group received conventional care, and the mother and baby were separated until the baby was clinically stable. The details of intervention and control arms and care of the newborns in both the groups are described in the already published protocol paper [11].

### Care of children in both intervention and control groups for the follow-up study

For the ongoing follow-up study, care of the children continues to be as per each site’s routine policy and standard of care, including routine health monitoring visits, care for acute illnesses, and management of morbidities including neurodevelopment impairments. This includes appropriate early diagnosis and management of the neurodevelopmental outcomes of interest. Children with suspicion of epilepsy are referred to appropriate neurologic or child health services for confirmation and management according to national protocols. Those with motor impairments are referred to local rehabilitation specialists for physical therapy as per local resource availability. For any child with signs of developmental impairment (motor, cognitive, or social-behavioral), referral to appropriate early stimulation programs is provided. Early monitoring for vision and hearing impairment is performed routinely and in case of any suspicion, the children are referred to appropriate ophthalmologic and audiology or otolaryngology specialists for appropriate management and to adapt to environment. For every child that has been identified as having neurodevelopmental impairment, each site in line with their standard protocol for the identification, assessment, management, and follow-up care develops an individual care plan (including rehabilitation, psychosocial support, etc.) in consultation with the child’s parents.

### Primary and secondary outcomes

The primary outcome of the follow-up study is the presence of neurodevelopmental impairments assessed by the age of 3 years in all enrolled children. The study duration and window for the last outcome assessment were extended from 2 to 3 years after ethics approval, due to the COVID-19 pandemic. Specifically, this composite outcome includes rates of motor impairment and risk of cerebral palsy, hearing impairment, vision impairment, cognitive, language, motor, socio-emotional or adaptive behavior, and epilepsy and the presence of any one or more will be considered as neurodevelopmental impairment. Secondary outcomes include growth and feeding practices, mortality and home environment (maternal depression and parent–child interactions), and rates of overnight hospital admissions.

Outcomes are assessed via standardized tools and questionnaires (Table 1). Motor impairment and risk of cerebral palsy are measured by standardized neurologic evaluation, using a validated tool, Hammersmith Infant Neurological Examination (HINE) at 6 months, 1 year, and 2 years of age. Cognitive, language, motor, socio-emotional, or adaptive behavior is assessed using Bayley Scales of Infant and Toddler Development (BSID III) at 2 to 3 years of age. Epilepsy is diagnosed using a standardized questionnaire, with operationalization of the International League against Epilepsy (ILAE) definition. Hearing is evaluated at discharge from the hospital or as soon as possible after enrolment in the follow-up study. First, an assessment by screening auditory brainstem responses (ABR) is performed at the facility. If the infant clears this assessment, no further assessment for hearing is required. In case the infant fails this initial screening, he/ she is referred to the audiologist or specialist for re-screening including a diagnostic ABR if required. Visual acuity is measured using Teller Acuity Cards, at or any time after 1 year of age at the facility.

**Table 1:** Outcome measurements

Outcomes	Tool	Measurement strategy	Rationale
**Primary outcome**			
Developmental delay	Bayley Scales of Infant and Toddler Development III (BSID III) The scale is used to assess language, cognitive, motor, social-emotional, and general adaptive functions	Assessment by Bayley-trained clinical psychologist/clinician between 24-and-36-month chronological age The BSID III scores will be calculated using corrected gestational age For the BSID III, the composite score range in different domains is as follows: Cognitive: 55–145 Language: 47–153 Motor: 46–154 Social-emotional: 55–145 Adaptive: 40–160 Composite score in each individual domain may be converted into scaled scores that range from 1 to 19. Average scaled scores are between 8 and 12 A composite score of < 80 will indicate developmental delay	BSID III has been developed in the USA and used in LMIC countries, including India [28–31] BSID III, in comparison to BSID II, requires a higher cutoff score for comparable sensitivity in detecting developmental delay. BSID III combined scores < 80 and cognitive and language scores < 85 were found to correlate with BSID II Mental Development Index (MDI) scores < 70 and clinically to moderate-severe developmental delay [31]
Cerebral palsy	Hammersmith Infant Neurological Examination (HINE)	Standardized neurological assessment using HINE at 6, 12, and 24 months According to a recent systematic review [32], HINE scores have 90% predictive accuracy of cerebral palsy By 12 months, a score of < 40 is predictive of non-ambulatory cerebral palsy [plegic, not walking] and a score of 40–60 is predictive of ambulatory cerebral palsy [able to walk], with any score < 70 considered abnormal. The maximum score is 78 The accepted norms will be used for the cutoff in this study, which are > 70 at 6 months and > 73 at 12 months and older [32–37] Due to the risk of more subtle findings, particularly in premature infants, there is increased sensitivity to the tool when used at multiple time points; therefore, the examination will be performed at an additional time point of 2 years For the primary outcome, the score at the 12- and 24-month assessments will be considered	HINE is a strongly recommended scale for early diagnosis of cerebral palsy [32] It is a standardized and scorable clinical neurological examination that is applicable for infants aged between 2 and 24 months and has been shown to be an easy-to-train tool with interobserver reliability even in less experienced staff [38]
Hearing impairment	Hearing assessment measured ideally at discharge or as soon as possible at enrolment or first follow-up	First assessment by screening auditory brainstem responses (ABR) If screening ABR is passed, no need for further hearing assessment tests In case screening ABR is failed, the participant is referred at the earliest possible opportunity to audiologist or specialist for re-screening, including, diagnostic ABR as applicable. Hearing impairment is defined as failed diagnostic ABR or failed re-screening ABR if diagnostic ABR is not available	International guidelines recommend hearing assessment within the first month of life whenever feasible [39] While Otoacoustic Emission (OAE) screening can be used in settings where ABR is not available, it is less preferred due to the potential for missing retro-cochlear hearing loss. Therefore, in studying high-risk infants, ABR is preferred [39–42]
Vision impairment	Structural visual examinations, including for retinopathy of prematurity, will be performed between discharge and 3 months, with visual acuity measured at 12 months	Visual acuity measured by Teller Acuity Cards at the facility Impairment defined by measurements 2 lines less than age-defined normative values	Teller Acuity Cards will be used due to the availability of age-specific reference ranges [43, 44]
Epilepsy	Screen for seizure at each follow-up visit. If screening positive, refer to a clinician	First assessment by epilepsy screening questionnaire at each follow-up visit If the participant screen negative, there is no need for further assessment tests In case the participant is screened positive, he/she will be assessed by a pediatrician/pediatric neurologist by a diagnostic questionnaire to arrive at a diagnosis of epilepsy/no epilepsy using the ILAE definition [45]. To reduce variability in diagnosis for purposes of this study, the ILAE diagnostic criterion has been operationalized for this study in the diagnostic questionnaire as either 2 unprovoked seizures separated by at least 24 h or 1 unprovoked seizure and one other positive finding of neurodevelopmental impairment	Screening questionnaires for pediatric epilepsy are limited due to small populations and limited age range and have demonstrated moderate validity [46, 47] A questionnaire comprised from validated pediatric epilepsy screening questionnaires and agreed upon by pediatric neurologists from the participating sites has been employed after local pilot testing
**Secondary outcomes**			
Mortality	Screen for death of enrolled child occurring any time between 29 days and 3 years of age	Mortality/survival status at each screening point. Assessments performed every 3 months until the age of 3 years	
Growth	Anthropometric parameters of weight (W), length (L), and head circumference (HC) for age using standardized equipment	Anthropometry at 6, 12, and 24 months of age. Parameters are plotted as per corrected age on WHO Multicentre Growth Reference Study (MGRS) charts and interpreted as per *z* scores Postnatal growth failure defined as fall in *z* scores or *z* scores < 2 SD	Measurements are obtained during facility visits. In case the participant is unable to visit the facility, we perform the measurement at the home of the participant
Feeding practices	Assessment of breastfeeding, expressed breast feeds, complementary feeds at 6 months, and adequacy of feeding beyond infancy	Feeding practices measured through a standard questionnaire at each follow-up visit—every 3 months until the age of 3 years	
Maternal depression	Patient Health Questionnaire 9 (PHQ-9)	Maternal depression measured using PHQ-9 at 6 and 12 months of age	Literature suggests, while early screening for post-partum depression is important, additional screening points at either 6 or 12 months can capture additional women who may have initially screened negative at earlier time points. Therefore, we have included these time points in this study [48]
Home environment	HOME	Assessment of home environment measured using standard HOME-SF scale at 18 months by a home visit	HOME is a validated tool across cultures. The HOME-SF scale has 6 subscales: (1) parental responsivity, (2) acceptance of the child, (3) organization of the environment, (4) learning materials, (5) parental involvement, and (6) variety in experience These align with the MAL-ED study which has also identified that a three-factor structure—emotional and verbal responsivity, clean and safe environment, and child cleanliness—can be examined cross-culturally [49]

The schedule of outcome assessments is shown in Table 2. As this is a follow-up study of the main iKMC trial participants, and was initiated in a phased manner in January 2019, approximately 14 months after the start of the parent trial, infants are enrolled on a rolling basis. While all efforts are made to recruit the infant within the first 3 months, participants from the parent trial who have completed 3 months of age prior to the follow-up study start date are recruited after the initial assessment time points. Assessments for outcome measurements are performed at the earliest age-appropriate time point feasible for the individual child upon enrolment for this follow-up study. Due to the challenge posed by the COVID-19 pandemic with ensuing lockdowns and permanent movement of many of the children of migrant laborers to distant areas, the age window for enrolment and completion of all assessments was extended to 36 months, and we switched to telephonic follow-up wherever we could but retained in-person follow-up for the primary outcome measurement at 12 and 24–36 months. This was implemented after due approval from the institutional review boards (IRBs) or ethics committees.

**Table 2:** Outcome measurement schedule

Assessment schedule	Discharge	3mo	6mo	9mo	1 yr	15mo	18mo	21mo	2 yr to 3 yr
Motor impairment/cerebral palsy risk			X		X				X
Hearing impairment	X								
Vision impairment					X				
Mental, motor, social-emotional development									X
Epilepsy		X	X	X	X	X	X	X	X
Mortality		X	X	X	X	X	X	X	X
Weight			X		X				X
Infant length/child height			X		X				X
Head circumference			X		X				X
Feeding practices		X	X	X	X	X	X	X	X
Hospitalization admission at least overnight		X	X	X	X	X	X	X	X
Home environment							X		
Maternal depression			X		X				
Approximate duration of the study visit	30 min	30 min	45 min	20 min	60 min	20 min	30 min	20 min	60–90 min

### Blinding

While the main iKMC trial evaluating the impact on newborn survival did not incorporate blinding due to the nature of the intervention, during the follow-up assessments, evaluators conducting the standardized assessments are blinded to whether the child received the intervention or not to decrease the bias.

### Study implementation strategy

The iKMC follow-up study is being conducted in a standardized manner across all the sites in the five countries. Each site has constituted dedicated multidisciplinary teams to perform the different activities—study conduct, internal quality control, project management, and data management. The conduct team responsible for consent as well as follow-up of the enrolled children for outcome measurements comprises research assistants, clinical psychologist, audiologist, and field workers. The investigators who are clinicians are responsible for overall study conduct and quality assurance and quality control. Each team member is trained in the protocol, Good Clinical Practice (GCP), and study-specific standard operating procedures specific to their role and responsibility. A site coordinator is responsible for coordinating the implementation of the follow-up study at each site. A central team led by WHO headquarters coordinates the conduct across all sites, ensures harmonization of processes, and monitors quality and study progress.

### Infrastructure

An important fallout of the study has been infrastructure development. Each site has developed a designated child development assessment unit. The unit has separate designated areas for registration and screening, consent, anthropometry, structured neurological assessment using HINE, developmental assessment using BSID III, visual acuity using Teller Acuity Cards, hearing assessment using auditory brainstem responses, and data entry and management.

### Enrolment and outcome measurements

The study conduct research staff is responsible for consent as well as follow-up of the enrolled children for outcome measurements.

They are blinded to the intervention or control group allocation of enrolled children as they were not involved with intervention delivery during the main trial conduct. The staff performing outcome assessments had an initial intensive training on the standardized and validated tools being used for assessing primary and secondary outcomes of the follow-up study described in Table 1. For any identified neurodevelopmental impairment, an appropriate standard of care management is provided to the child.

### Quality assurance and quality control

The study has a well-structured quality assurance plan that is being implemented at all sites. The site coordinators ensure adherence to the manual of operations. The study has employed several checks to ensure the quality of all aspects of the follow-up study including the consent process and the outcome measure assessments. Internal quality checks are conducted by the site coordinators and study principal investigators (PIs) at each site. The data acquisition for the outcome measurements is verified by the site coordinator. Ten percent of the data acquisition is reviewed by the expert investigator/designee for completeness and consistency. The investigators monitor at least 10% of processes being performed for different outcome measurements by the study conduct team. The observations are documented in specifically designed forms. Appropriate corrective and preventive actions are taken based on expert observations. External oversight and support is provided by WHO headquarters to ensure the quality of study implementation. This is done through site visits by WHO staff or consultants using a standardized monitoring checklist. Additionally, sites transfer data to WHO headquarters every month and this data is reviewed by the central team at WHO for quality, and feedback for improvement is provided to the PIs at respective sites.

### Training and standardization

The research staff including the conduct team, project management team, data management, and internal quality control team underwent an intensive training before the start of the study. All were oriented to the protocol and GCP guidelines. Orientation and sensitization of regular health care staff of the Departments of Pediatrics, Physical Medicine and Rehabilitation, Ophthalmology and Ear, Nose and Throat regarding the study and its processes was done to ensure smooth functioning. The research staff has been provided role-specific intensive training and standardization. The research assistants and coordinators from the conduct team were trained on screening for eligibility, administering written informed consent from the parents, enrolment, outcome measure assessments, and scheduling and tracking for the 3-year follow-up of the children to minimize deviations and non-compliance to the follow-up time points. All research staff were trained in rapport building and communication with the mother and the families. Training for specific outcome measurements like visual acuity using Teller Acuity Cards, hearing using screening ABR, and maternal depression using the 9-question patient health questionnaire (PHQ-9) was provided to the designated staff. There was a 2-week intensive training for Bayley’s assessment by experts. A training of trainers (TOT) workshop for standardization of anthropometry measurements of length, head circumference, and weight was conducted by experts and the Safdarjung hospital, India investigators for all the five study sites. Each site was trained in the implementation of each tool, counseling of families if a neurodevelopmental impairment is identified with an appropriate referral system in place, and training local providers at each site.

All participating hospitals are supported to make quality-of-care improvement and provide standard of care with breast milk feeding support, complementary feeding support, and attention to hygiene for all infants.

### Data collection

The data for the study is being collected on paper-based case report forms which are transcribed into an electronic database. The electronic database has been developed on a clinical data management platform “REDCap” with all the data quality checks. The questions, response options, variable names, and data structure are identical for all sites. Double data entry is done at each site by trained entry personnel. The data manager at each site is responsible for data quality checks, query management, and ensuring completeness of data. Any discrepancies are addressed within 24 h of data collection. All data collected is password protected and stored in a local server in each site. No personal identifiers are entered in the database. All paper forms are stored in locked filing cabinets at the respective sites.

### Data management and analysis

The data management is coordinated centrally by a team from WHO headquarters, but each site is responsible for site data management and data security. A central data repository has been created at the WHO headquarters. Sites share cleaned data every month to WHO where additional checks are run and a list of queries sent to the sites for clarification. All data sent to WHO does not have any personal identifiers.

The data from this multicenter study will be accessible to all the participating research teams to jointly answer the study questions. After the publication of the manuscript reporting the results on primary and secondary outcomes, the data will be made publicly accessible.

### General principles for analysis

The analysis will be by intention to treat. The primary outcome is a composite outcome comprising cerebral palsy, hearing impairment, vision impairment, cognitive, language, socio-emotional, adaptive and motor impairment, and epilepsy, and the presence of any one or more will be considered as neurodevelopmental impairment. However, during analyses, we will also consider each of these outcomes individually. Table 1 includes the scale/tool used for outcome measurement and wherever appropriate, the cutoffs of the scales, to indicate the presence or absence of the neurological impairment.

The primary and secondary outcomes will be compared between the intervention and control groups. All planned analysis will use a 5% significance level. Risk ratios and their confidence intervals will be calculated and will be the primary analysis if the loss to follow-up is 2.5% or lower. In case the loss to follow-up for the primary outcome is greater than 2.5%, we will additionally calculate hazard ratios and their confidence intervals. Additionally, we will adjust the results for confounding using multiple logistic regression and Cox proportional hazards models if there are any important differences in baseline characteristics between immediate KMC and control groups.

If more than 5% of data is missing from interim assessments, multiple imputation will be performed, accounting for clustering by country with the number of imputations based on the percentage of missing data. Subgroup analysis will be conducted by (i) birth weight categories (1 to < 1.2 kg, 1.2 to < 1.5 kg, and 1.5 to < 1.8 kg), (ii) gestation (< 34, 34–36, >=37 weeks), and (iii) singleton/multiple birth. Secondary analysis will be limited to an analysis by compliance. This secondary analysis will present the efficacy of the intervention by average duration of skin-to-skin contact over the first 3 days of life, categorized as > =20 h/day, 10–19 h/day, and < 10 h/day.

### Study oversight

The study Steering Committee comprises all PIs from study sites, Bill and Melinda Gates Foundation representatives, and WHO technical staff functioning as its secretariat. This committee is responsible for designing and implementing the study in a harmonized way. Study PIs are responsible for contributing to the development of the research proposal, study manual, data management system, outcome measurement and data collection, data analysis and interpretation, and dissemination of results. All activities are facilitated and supported by WHO headquarters. On a fortnightly basis, the sites submit a brief status report to WHO. A formal progress report is submitted by each site every year.

The study is coordinated by a technical team from WHO headquarters, ensuring arrangements are in place to support teams in any challenges being faced to implement this study. WHO technical staff conducted intensive training at the beginning of the study. The technical team performs monitoring visits to each site every year. Monitoring visits have the dual function of identifying problems and supporting the sites in improving data collection, follow-up, and monitoring.

A Technical Advisory Group (TAG) has been set up in the field. The TAG members serve in their individual capacity and reviewed the final research protocol for any major concern prior to trial implementation. TAG members’ terms of reference also include revision of manual of operations, study forms, and consent forms and advise on practical issues in implementing the trial in the field.

There is no data and safety monitoring board constituted for this study as this is a prospective follow-up study of a cohort of trial participants.

## Discussion

This international multi-site study is the first of its kind aiming to assess the effect of immediate KMC on long-term outcomes. The literature has demonstrated an immediate benefit of KMC in stable neonates and is part of WHO guidelines for newborn care [5, 6]. More recently, the main iKMC trial showed that the benefits of KMC can be extended to unstable low birth weight babies, beginning right at birth, and iKMC had a 25% lower risk of death at 28 days than those who received conventional care with Kangaroo Mother Care initiated after stabilization [12]. With the parent trial successfully conducted across these sites, there is no better opportunity to study the effect of this intervention on long-term outcomes. The follow-up study has the objective of elaborate and precise assessment of all the domains of neurodevelopment, including cerebral palsy (HINE), developmental delay (BSID III), hearing, vision, and epilepsy screening which would be evaluated by trained personnel ensuring optimal standardization on the large sample size in the five LMIC countries. The training and standardization conducted as part of this study have already strengthened the infrastructure and supported capacity building in the participating sites. This can be scaled up in the future at various tertiary centers in LMICs, in order to ensure quality, follow-up care for each and every mother-NICU/NICU graduate.

In addition, the final results will provide crucial insights and a higher level of evidence for the much-awaited long-term outcome of immediate KMC in the vulnerable sick low-birth-weight neonates. If proven effective, this intervention would be a value addition to the set of cost-effective strategies in reducing neurological impairment in resource-limited countries.

### Study status

The trial is ongoing in all five sites—Ghana, India, Malawi, Nigeria, and Tanzania. The first participant was recruited on 11 January 2019. Participant recruitment and completion of all assessments are expected to be completed by 20 July 2022. The current protocol is version 3.2 dated 7 April 2021.

## Data Availability

The datasets generated during the current study will be available from the corresponding author on reasonable request.

## Abbreviations

KMC: Kangaroo Mother Care
iKMC: Immediate Kangaroo Mother Care
LIMC: Low- and middle-income income countries
LBW: Low birth weight
WHO: World Health Organization
RCT: Randomized controlled trial
HINE: Hammersmith Infant Neurological Examination
BSID III: Bayley Scales of Infant and Toddler Development
ILAE: International League against Epilepsy
ABR: Auditory brainstem responses
IRB: Institutional Review Board
GCP: Good Clinical Practice
PI: Principal investigator
PHQ-9: Patient Health Questionnaire-9
TOT: Training of trainers
TAG: Technical Advisory Group
